# Unveiling the Power of Bergamot: Beyond Lipid-Lowering Effects

**DOI:** 10.3390/nu17111871

**Published:** 2025-05-30

**Authors:** Myriam Carpenito, Federica Coletti, Saverio Muscoli, Lorenzo Guarino, Anna Di Cristo, Valeria Cammalleri, Simona Mega, Sara Emerenziani, Michele Cicala, Chiara Fanali, Gian Paolo Ussia, Francesco Grigioni

**Affiliations:** 1 Department of Cardiovascular Sciences, Università Campus Bio-Medico di Roma, Via Alvaro del Portillo, 21, 00128 Rome, Italy; m.carpenito@policlinicocampus.it (M.C.); anna.dicristo@unicampus.it (A.D.C.); g.ussia@policlinicocampus.it (G.P.U.); f.grigioni@policlinicocampus.it (F.G.); 2 Fondazione Policlinico Universitario Campus Bio-Medico, 00128 Rome, Italy; federica.coletti@unicampus.it (F.C.); lorenzo.guarino@policlinicocampus.it (L.G.); v.cammalleri@hotmail.it (V.C.); s.mega@policlinicocampus.it (S.M.); 3 Division of Cardiology, Policlinico Tor Vergata, Viale Oxford 81, 00133 Rome, Italy; 4 Gastroenterology Unit, Campus Bio-Medico University of Rome, Via Álvaro del Portillo 21, 00128 Rome, Italy; s.emerenziani@policlinicocampus.it (S.E.); m.cicala@policlinicocampus.it (M.C.); 5 Department of Science and Technology for Sustainable Development and One Health, Università Campus Bio-Medico di Roma, Via Alvaro del Portillo 21, 00128 Rome, Italy; c.fanali@unicampus.it

**Keywords:** cardiovascular diseases, dyslipidemia, nutraceuticals, bergamot

## Abstract

Dyslipidemia is a leading risk factor for cardiovascular diseases. Nutraceuticals for the management of dyslipidemia are gaining growing attention. Derived from food sources, they represent a promising adjunctive or alternative therapeutic option in specific clinical contexts, particularly in individuals with mild dyslipidemia or those who fail to achieve lipid targets despite optimal pharmacological treatment. Bergamot—alongside its lipid-lowering effects—has demonstrated multiple additional properties, including anti-inflammatory, antioxidant, and vascular benefits. While lipid effects are supported by several clinical studies, evidence for vascular and inflammatory pathways is based mainly on preclinical studies. This review summarizes the mechanisms of action and available clinical evidence and outlines potential indications for bergamot use in selected patient subgroups.

## 1. Introduction

Cardiovascular diseases (CVDs) are the leading cause of morbidity and mortality worldwide, primarily driven by a combination of non-modifiable (gender and family history) and modifiable (e.g., dyslipidemia, hypertension, smoking, diabetes, and obesity) risk factors. Among modifiable risk factors, low-density lipoprotein cholesterol (LDL-C) is recognized as the most significant contributor to atherosclerosis [1,2,3]. An undisputable body of evidence demonstrates a linear relationship between LDL-C and cardiovascular risk reduction [2]. Lifestyle interventions are crucial to manage dyslipidemia in those with low cardiovascular risk [3]. For instance, adopting a Mediterranean diet, engaging in regular physical activity, and maintaining a healthy weight can reduce LDL-C by 5% to 15% [4]. Besides lifestyle modifications, pharmacological treatments remain pivotal due to their high efficacy and effectiveness. These include statins, ezetimibe, and novel therapies such as proprotein convertase subtilisin/kexin type 9 (PCSK9) inhibitors, small interfering RNA (siRNA), and bempedoic acid [5]. Nutraceuticals derived from food sources offer a promising adjunctive or alternative therapeutic option for individuals with mild dyslipidemia or those who fail to achieve lipid targets despite optimal pharmacological treatment [6]. This narrative review examines the effects of bergamot on cardiovascular health, summarizing its mechanisms of action and the most relevant preclinical and clinical evidence. In contrast to previous reviews, which have primarily focused on lipid modulation, this work integrates anti-inflammatory, antioxidant, and endothelial effects, outlining its full range of pleiotropic profiles. We included peer-reviewed original studies and meta-analyses published in English between January 2010 and December 2024. Studies involving in vitro, animal, and human models were considered. Non-peer-reviewed articles and studies with incomplete outcome data were excluded. As a narrative review rather than a systematic one, we had no predetermined research questions or specified protocols. Disagreements were resolved by discussion with other team members.

## 2. Bergamot as a Source of Flavonoids

**Figure 1:**
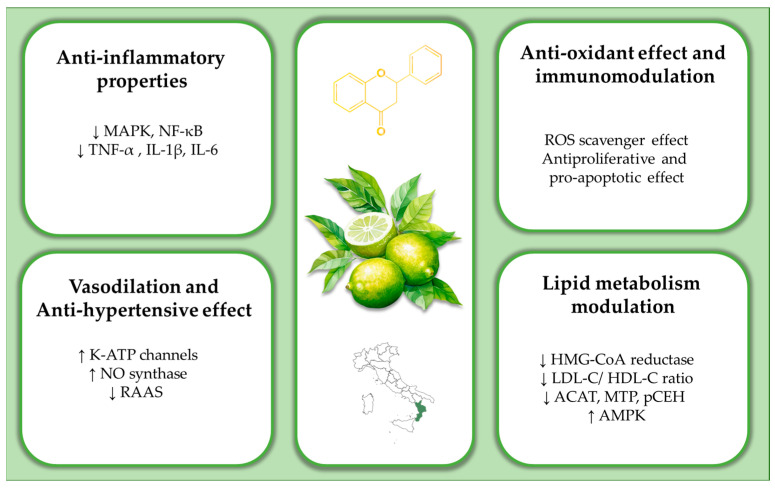
Pleiotropic effects of bergamot. MAPK, mitogen-activated protein kinase; NF-kB, nuclear factor kappa-light-chain-enhancer of activated B cells; TNF- α, tumor necrosis factor-α; IL-1β, interleukin-1β, IL-6, interleukin-6; ROS, reactive oxygen species; K-ATP channels, adenosine triphosphate-sensitive potassium channels; NO, nitric oxide; RAAS, renin–angiotensin–aldosterone system; HMG-CoA reductase, 3-hydroxy-3-methylglutaryl coenzyme A reductase; LDL-C, low-density lipoprotein cholesterol; HDL-C, high-density lipoprotein cholesterol; ACAT, acyl-CoA cholesteryl acyltransferase; MTP, microsomal triglyceride transfer protein; pCEH, pancreatic cholesterol ester hydrolase; AMPK, adenosine monophosphate-activated protein kinase. ↑ increased; ↓ decreased.

Flavonoids, a well-known group of polyphenols, are increasingly recognized for their potential protective effects on the cardiovascular system. Their anti-inflammatory and antioxidant properties may reduce the risk of CVD [7]. Flavonoids are small molecules characterized by a typical carbon structure (C6-C3-C6) consisting of two aromatic rings (A and B) and a heterocyclic ring (C), commonly referred to as the “flavan skeleton”. Variations in oxidation and substitution patterns on this core structure give rise to several subclasses, including flavones (e.g., luteolin and apigenin), flavonols (e.g., quercetin and kaempferol), flavanones (e.g., naringenin and hesperetin), flavan-3-ols (e.g., epicatechin and epigallocatechin-3-gallate), anthocyanidins, and isoflavones (e.g., daidzein and genistein) [7]. These compounds are abundant in foods such as tea, red wine, citrus fruits, olive oil, and aromatic herbs and spices. Dietary flavonoid intake varies widely, ranging from 50 to 800 mg per day, depending on individual nutritional practices [8]. Bergamot (Citrus bergamia Risso), a citrus fruit native to southern Italy, particularly the Calabria region, is renowned for its essential oil (BEO), extracted from the peel via cold pressing. Traditionally, the bergamot juice (BJ) obtained by pressing the remaining fruit pulp was considered a waste byproduct of the essence industry. However, over the past decade, BJ has garnered significant scientific interest as a rich source of bioactive compounds [9,10]. The Bergamot Polyphenolic Fraction (BPF)—a purified extract derived from BJ through solvent extraction and chromatographic techniques—contains particularly high concentrations of flavanones such as naringin (up to 400 mg/L), neoeriocitrin (~250 mg/L), and neohesperidin (~150 mg/L), which are rare or absent in other citrus fruits like lemons and oranges [9,10]. These compounds have been demonstrated to enhance lipid metabolism and improve endothelial function in both preclinical and clinical studies. The main cardiovascular effects of bergamot are discussed below and summarized in Figure 1.

### 2.1. Anti-Oxidant and Anti-Inflammatory Properties

Flavonoids exhibit significant anti-inflammatory effects, as demonstrated in both in vitro and in vivo studies [11,12,13]. A flavonoid-rich extract from BJ has been shown to reduce pro-inflammatory cytokines, including tumor necrosis factor-alpha (TNF-α), interleukin-1β (IL-1β), and interleukin-6 (IL-6), as measured by enzyme-linked immunosorbent assay (ELISA) and confirmed at the gene expression level using quantitative real-time polymerase chain reaction (qRT-PCR) [12,13,14]. This extract also attenuates the activation of nuclear factor kappa B (NF-κB) and the phosphorylation of mitogen-activated protein kinase (MAPK), resulting in reduced inflammatory tissue damage in animal models [14]. In addition, bergamot flavonoids enhance antioxidant defenses by scavenging reactive oxygen species (ROS), thereby mitigating oxidative stress and promoting cellular homeostasis [15].

### 2.2. Effects on Vascular Endothelium

The vascular effects of bergamot have been demonstrated in both in vitro and in vivo models [16,17,18,19,20]. Bergamot flavonoids support endothelial function by promoting vasodilation and regulating blood pressure through mechanisms such as the activation of calcium and ATP-sensitive potassium channels, as well as by enhancing nitric oxide (NO) bioavailability [16,17,18,19,20,21]. Compounds such as neohesperidin, naringin, and rutin help preserve endothelial integrity and reduce vascular injury [22,23,24]. In murine models, neohesperidin has been shown to inhibit angiotensin II-induced vascular remodeling and oxidative stress by downregulating nicotinamide adenine dinucleotide phosphate (NADPH) oxidase and inflammatory cytokines, as assessed by immunohistochemistry and ELISA [25,26]. Moreover, dietary supplementation with bergamot polyphenols mitigated hypertension-induced cardio-renal damage in a rat model of unilateral renal artery ligation [27].

### 2.3. Effects on Lipid Metabolism

Bergamot extract has emerged as a promising natural compound with potential lipid-lowering properties [28,29]. One of its primary mechanisms is the inhibition of 3-hydroxy-3-methylglutaryl coenzyme A (HMG-CoA) reductase, the key enzyme in cholesterol biosynthesis, thereby mimicking the action of statins. The BPF contains two unique compounds—brutieridin and melitidin—which bind directly to the enzyme’s active site and effectively improve the LDL-C/high-density lipoprotein cholesterol (HDL-C) ratio [30,31,32]. In vitro studies using HepG2 hepatoma cells demonstrated that these compounds reduced intracellular cholesterol synthesis by 30–40% [30,31]. Bergamot also contributes to triglyceride (TG) reduction by inhibiting acyl-CoA cholesteryl acyltransferase (ACAT) and microsomal triglyceride transfer protein (MTP)—two enzymes involved in lipoprotein assembly [33,34]. In addition, bergamot activates AMP-activated protein kinase (AMPK-α), which in turn inhibits sterol regulatory element-binding protein-1 (SREBP-1), thereby further reducing cholesterol and TG synthesis [35,36]. Preclinical studies support these mechanisms, confirming the lipid-lowering potential of bergamot in animal models [37,38,39]. BPF has also been shown to promote cholesterol elimination by inhibiting pancreatic cholesterol ester hydrolase (pCEH), thereby enhancing bile acid synthesis and fecal sterol excretion [40]. In hyperlipidemic rats, daily administration of 10 mg/kg BPF significantly reduced LDL-C, TG, and PCSK9 levels while increasing HDL-C [41]. Higher doses (e.g., 50 mg/kg) led to greater reductions in cholesterol and TG, particularly in animals on a hyperlipidemic diet, suggesting that dietary factors may influence BPF effectiveness [42,43]. These results were comparable to those obtained with red yeast rice [44].

#### 2.3.1. Clinical Evidence

Clinical evidence corroborates preclinical findings, demonstrating that bergamot supplement reduces lipid levels when administered at 200 to 1500 mg/day [45]. However, results across trials vary significantly due to differences in participant characteristics, dosage regimens, extraction methods, and the presence of additional bioactive compounds. A 30-day randomized trial demonstrated that 500 mg/day of BPF significantly reduced total cholesterol (−21.8%), LDL-C (−24.1%), and triglycerides (TG) (−30.5%) compared to placebo in individuals with hyperlipidemia [44]. Similarly, a daily dose of 500 mg of a formulation containing bergamot fruit extract, soy, berberine, and phytosterols reduced LDL-C by 18.8% and TG by 50.8% when combined with a low-glycemic Mediterranean diet [45]. In patients with mixed hyperlipidemia, 500 mg/day of bergamot extract, combined with plant sterols and vitamins, produced a 7.63% reduction in LDL-C compared to the placebo [46]. Higher doses of bergamot, as a single active compound (1000 mg/day), lead to reductions of 31.3% in total cholesterol, 40.8% in LDL-C, and 30.7% in TG- effects comparable to those of low-dose rosuvastatin [41]. Notably, bergamot has also been shown to enhance the impact of reduced-dose statins in combination therapies [47,48]. Long-term administration of 1300 mg/day of BPF over 120 days in patients with metabolic syndrome resulted in reductions of 25.7% in total cholesterol, 37.7% in LDL-C, and 31.0% in TG [49,50,51]. A nutraceutical blend containing 375 mg of bergamot dry extract and artichoke also improved fasting glucose, LDL-C, TG, and HDL-C levels, as well as inflammatory markers such as C-reactive protein [52]. These findings confirm the dose-dependent lipid-lowering effects of bergamot and underscore its synergistic potential when used in combination with statins or other bioactive compounds. A summary of the key clinical studies is provided in Table 1.

#### 2.3.2. Pharmacological Data

The main active compounds in bergamot, such as melitidin and brutieridin, share structural features with the HMG component of statins, which may explain their affinity for HMG-CoA reductase and contribute to their cholesterol-lowering properties [44]. Despite encouraging preclinical and clinical data, pharmacokinetic information on bergamot flavonoids in humans remains limited. Like other citrus flavonoids, these compounds are thought to undergo intestinal and hepatic metabolism, with glucuronidation and sulfation likely affecting their bioavailability [31]. Absorption and elimination kinetics are still poorly defined, partly due to the variability among commercial formulations, which differ in flavonoid content, degree of standardization, and concentration of active ingredients. In animal studies, repeated oral administration of BPF at doses up to 150 mg/kg/day did not produce evidence of hepatotoxicity, nephrotoxicity, or hematologic toxicity [41]. Human trials have generally reported good tolerability, with no significant adverse events observed during treatment periods of up to 120 days [47]. However, data on chronic exposure, higher doses, and long-term safety remain scarce.

## 3. Outline for Bergamot Supplementation in Clinical Practice for Dyslipidemia

### 3.1. “Who” Are the Most Appropriate Candidates?

#### 3.1.1. Statin-Intolerant Patient

Statins are widely recognized as the first-line treatment for dyslipidemia in individuals at moderate to very high cardiovascular risk [53,54,55]. However, high-dose or high-intensity statins are frequently associated with adverse effects, particularly muscle-related symptoms such as myalgia, myopathy, and, in severe cases, rhabdomyolysis [53]. Statin intolerance is relatively common, with estimates suggesting that between 45,000 and 290,000 individuals may be affected annually [54]. This condition poses a significant barrier to treatment adherence and often fails to achieve the LDL-C target [55]. Recent investigations have kindled hope by exploring the potential of nutraceuticals as alternative or adjunctive interventions for patients experiencing statin-associated muscle symptoms. Among them, bergamot extract has shown promise in early studies. In an open-label study involving 237 patients with isolated hypercholesterolemia, mixed dyslipidemia, or metabolic syndrome, bergamot extract administered at 500 mg/day and 1000 mg/day significantly improved lipid profiles. In statin-intolerant patients, a dose of 1500 mg/day following a 60-day washout also yielded favorable results, with no reported adverse effects [44]. These findings have led to an increasing interest in nutraceuticals as adjunctive tools in managing statin intolerance. Although further validation is needed, some expert consensus statements have acknowledged the potential role of bergamot in selected patients who do not tolerate conventional lipid-lowering therapies. Despite this promising evidence, the journey is far from over. Larger cohorts of statin-intolerant patients, extended follow-up periods, and evaluations of cardiovascular outcomes are essential to confirm these findings and establish long-term clinical benefits. Moreover, large-scale randomized clinical trials specific to this population are needed to verify and reinforce these findings.

#### 3.1.2. Patients at Low Cardiovascular Risk

In many countries, the decision to initiate statin therapy is based on a 10-year cardiovascular (CV) risk estimation, which incorporates demographic, clinical, and biomarker data. However, this short-term model may underestimate the lifetime CV risk of younger individuals with isolated dyslipidemia and no additional risk factors [56]. Concerning LDL-C levels, extensive epidemiological and clinical evidence supports the principle that “less is better for longer”, emphasizing the cumulative benefits of sustained LDL-C reduction over time. Although nutraceuticals typically result in only mild reductions in LDL-C levels compared to conventional lipid-lowering therapies, even small, sustained decreases in LDL-C can significantly lower cardiovascular risk if maintained over time. Initial management in low-risk patients should prioritize lifestyle interventions, including regular physical activity, weight loss, smoking cessation, and adherence to a diet rich in plant-based proteins and fiber and low in saturated fats. These measures remain the cornerstone of dyslipidemia management. Nutraceuticals may serve as an adjunctive strategy in cases where LDL-C levels stay above 116 mg/dL despite optimal lifestyle modifications [57]. This approach aligns with a preventive model that integrates behavioral and nutraceutical interventions to achieve durable cardiovascular protection over the life course.

#### 3.1.3. Optimization of Therapy in High-Risk Patients

**Figure 2:**
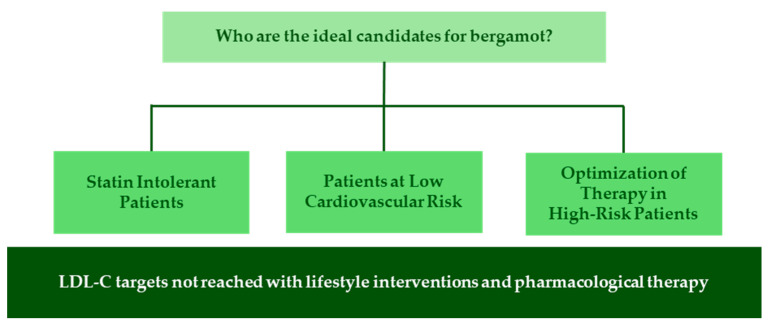
Clinical profiles for potential bergamot supplementation in dyslipidemia management. LDL-C, low-density lipoprotein cholesterol.

In high-risk patients receiving standard lipid-lowering therapies who are near but have not yet achieved their LDL-C targets, nutraceuticals may offer a valuable adjunctive strategy (Figure 2). Bergamot represents a promising addition to such regimens due to its statin-like effects and its ability to promote cholesterol excretion through mechanisms distinct from those of PCSK9 inhibitors and ezetimibe [44]. Beyond its lipid-lowering action, bergamot exhibits vasoprotective and anti-inflammatory properties that align with the therapeutic goals in high-risk patients, including improved endothelial function and reduced oxidative stress [58]. Evidence suggests a potential additive or synergistic effect when bergamot is combined with first-line pharmacological agents. For instance, Gliozzi et al. [47] demonstrated that co-administration of BPF with rosuvastatin significantly improved lipid profiles compared to rosuvastatin alone, accompanied by reductions in biomarkers of oxidative vascular injury. Preliminary studies suggest that bergamot is safe and well-tolerated, making it an attractive candidate for combination therapy [44,59]. Nevertheless, rigorous randomized clinical trials are needed to confirm its efficacy and define its role within lipid-lowering treatment algorithms. Additionally, data on potential adverse effects or pharmacodynamic interactions are still lacking. Clinicians should, therefore, remain cautious when introducing bergamot in patients receiving polypharmacy and monitor for possible drug–nutraceutical interactions.

### 3.2. “What” Do Position Papers Recommend?

Recent clinical trials, cohort studies, and meta-analyses underscore the potential of nutraceuticals in managing dyslipidemia and preventing cardiovascular disease. The International Lipid Expert Panel (ILEP) has released a position paper summarizing the evidence supporting the lipid-lowering effects of selected nutraceuticals and providing consensus-driven recommendations for their clinical use [60]. Each nutraceutical was evaluated based on the strength of recommendation and level of supporting evidence. Bergamot received a Class IIa recommendation with Level B evidence, indicating its demonstrated ability to reduce LDL-C levels by approximately 15% to 35–40% in clinical studies using doses ranging from 500 to 1500 mg/day. These studies also reported a favorable safety profile with no significant adverse effects. In a subsequent position paper, the ILEP explored the anti-inflammatory properties of nutraceuticals, acknowledging the central role of systemic inflammation in the pathogenesis of atherosclerotic CVD [61]. Preclinical data suggest that bergamot possesses promising anti-inflammatory activities [62,63]. For instance, in models of periodontal disease, bergamot reduced tissue damage and markers of gingival inflammation [14], while in models of inflammatory bowel disease, it decreased pro-inflammatory cytokine levels and apoptosis [64]. However, due to the limited availability of human data, the ILEP assigned bergamot a Class III recommendation with Level C evidence for its anti-inflammatory effects [65].

A more recent expert consensus further emphasized the therapeutic potential of nutraceuticals as an alternative or adjunctive strategy for managing statin intolerance [66]. Recommendations include the use of nutraceuticals as monotherapy or in combination in high- or very-high-risk patients with partial statin intolerance who fail to reach LDL-C goals with tolerable statin therapy and/or non-statin therapy (Class IIa, level B). Nutraceuticals are also recommended in intermediate- or low-risk patients with statin intolerance and elevated LDL-C who have not reached LDL-C targets (Class IIa, level A). Specifically, Bergamot extract received a Class IIb recommendation, based on level B evidence, for its use in statin-intolerant individuals [66].

International guidelines provide a mixed perspective. The 2019 guidelines from the European Society of Cardiology (ESC) and the European Atherosclerosis Society (EAS) [67] acknowledge the role of specific dietary supplements—such as red yeast rice, omega-3 fatty acids, and phytosterols—in individuals who are not candidates for statins based on global CV risk or as adjunctive agents in high- and very-high-risk patients who have not achieved LDL-C goals. Conversely, the 2019 American College of Cardiology (ACC) and American Heart Association (AHA) guidelines for primary prevention and cholesterol management do not provide specific recommendations regarding nutraceuticals [68]. This discrepancy likely reflects differing methodological approaches. The ACC/AHA guidelines prioritize recommendations supported by evidence from large randomized controlled trials, which ensures a high threshold for clinical adoption. In contrast, the ESC/EAS guidelines adopt a broader evaluative framework that incorporates expert consensus and real-world evidence, especially in areas where large-scale trials are lacking.

### 3.3. “How” Can Bergamot Be Supplemented in Clinical Practice?

According to the body of evidence examined in this review, bergamot has been administered in clinical studies at daily doses ranging from 150 mg to 1500 mg. Evidence suggests a dose-dependent relationship between bergamot intake and lipid-lowering efficacy. A systematic review by Lamiquiz-Moneo et al. [69] reported that higher doses are associated with greater reductions in total cholesterol, LDL-C, and TG. However, despite this trend, lipid-lowering responses vary substantially across studies. For instance, Cai et al. [46] observed a 7.63% reduction in LDL-C with 500 mg/day of whole-bergamot extract, whereas Mollace et al. [44] reported a 24.1% reduction using the same dose of standardized BPF. In contrast, Toth et al. [70] documented an 18.2% LDL-C reduction with 150 mg/day of isolated bergamot flavonoids. These differences could arise from variations in participant characteristics, bergamot formulation, and administration methods, underscoring the necessity for standardized protocols in future research.

In clinical practice, bergamot supplementation should be guided by patient phenotype, risk profile, and therapeutic goals. Available human trials, lasting up to 120 days, have reported no serious adverse effects. However, long-term safety data remain limited [44,46,69,70]. Ideal candidates for bergamot supplementation include patients with statin intolerance, those at low to intermediate cardiovascular risk who have not achieved LDL-C targets through lifestyle interventions alone, and high-risk individuals who require additional lipid-lowering without escalation of pharmacologic therapy. Clinicians should nonetheless be aware of the variability in commercial formulations and the lack of standardization in active compound concentrations.

Despite encouraging results, several limitations remain. The available studies vary considerably in terms of dosage, extraction techniques, and study populations, making it difficult to compare outcomes or draw consistent conclusions. Differences in formulation quality and individual metabolic responses add further uncertainty. Well-conducted human studies are still needed to clarify pharmacokinetics, confirm biological mechanisms, and guide appropriate use in practice.

## 4. Conclusions

This review offers an updated synthesis of current evidence on bergamot as a nutraceutical with potential relevance in the context of dyslipidemia and cardiovascular prevention. Alongside its lipid-lowering properties, bergamot appears to exert antioxidant, anti-inflammatory, and endothelial effects, acting on multiple pathways involved in lipid metabolism, vascular function, and systemic inflammation. Clinical studies suggest a dose-related impact on lipid parameters. While data from preclinical studies are encouraging, larger randomized trials with standardized formulations are needed to establish long-term safety, optimal dosing, and real-world efficacy across different patient populations.

## Figures and Tables

**Table 1:** Summary of clinical studies evaluating the lipid-lowering effects of bergamot.

	Population	Study Design	Dosage	Main Results
**Mollace et al., 2011** [44]	237 patients with hyperlipidemia ± hyperglycemia	Open-label study	500 mg/day	↓ LDL-C, TG, glucose; ↑ HDL-C; HMG-CoA inhibition; ↑ vasodilation
**Dahlberg et al., 2017** [45]	44 overweight patients with cardiometabolic syndrome	RCT	500 mg/day	↓ LDL-C, TG, ApoB/ApoA1, hs-CRP; ↓ CVD risk score
**Cai et al., 2017** [46]	98 patients over 65 years old with hyperlipidemia	RCT, double-blind, placebo-controlled	500 mg/day	↓ LDL-C, TG; ↑ HDL-C; ↓ weight, waist, BMI
**Gliozzi et al., 2013** [47]	77 patients with hyperlipidemia	RCT, open-label	1000 mg/day	↓ Total-C, LDL-C, LDL/HDL; ↓ oxidative stress markers
**Campolongo et al., 2016** [48]	64 patients with hyperlipidemia treated with simvastatin	Observational study	200 mg/day	↓ Muscular side effects from statins
**Gliozzi et al., 2014** [49]	107 patients with cardiometabolic syndrome	RCT, open-label	650 mg twice a day	↓ LDL-C, TG, glucose; ↑ HDL-C; ↓ NAFLD biomarkers
**Babish et al., 2016** [50]	11 patients with moderate dyslipidemia	Pilot observational study	250 mg/day	↓ LDL-C, non-HDL-C, ApoB; improved lipid ratios
**Mollace et al., 2019** [51]	60 patients with T2DM and hyperlipidemia	Open-label trial	650 mg twice a day	↓ LDL-C, TG, glucose; ↑ HDL-C; ↓ small dense LDL
**Fogacci et al., 2024** [52]	90 healthy individuals with suboptimal cholesterol	RCT, double-blind, placebo-controlled	375 mg/day	↓ Total-C, LDL-C, TG, non-HDL-C, hs-CRP, ApoB-100

LDL-C, low-density lipoprotein cholesterol; TG, triglycerides; HDL-C, high-density lipoprotein cholesterol; HMG-CoA reductase, 3-hydroxy-3-methylglutaryl coenzyme A reductase; RCT, randomized clinical trial; hs-CRP, high sensitivity C-reactive protein; BMI, body mass index; oxyLDL, oxidized low-density lipoprotein; total-C, total cholesterol; NAFLD, non-alcoholic fatty liver disease; T2DM, type 2 diabetes mellitus. ↑ increased; ↓ decreased.

## Data Availability

The original data presented in the study can be found in the bibliographic references.
